# Resequencing of durian genomes reveals large genetic variations among different cultivars

**DOI:** 10.3389/fpls.2023.1137077

**Published:** 2023-02-16

**Authors:** Wanapinun Nawae, Chaiwat Naktang, Salisa Charoensri, Sonicha U-thoomporn, Nattapol Narong, Orwintinee Chusri, Sithichoke Tangphatsornruang, Wirulda Pootakham

**Affiliations:** ^1^ National Omics Center (NOC), National Science and Technology Development Agency (NSTDA), Thailand Science Park, Pathum Thani, Thailand; ^2^ Chantaburi Horticulture Research Center, Horticulture Research Institute, Department of Agriculture, Chantaburi, Thailand

**Keywords:** durian, genome assembly, pangenome, comparative genomics, genomic structural variation

## Abstract

Durian (*Durio zibethinus*), which yields the fruit known as the “King of Fruits,” is an important economic crop in Southeast Asia. Several durian cultivars have been developed in this region. In this study, we resequenced the genomes of three popular durian cultivars in Thailand, including Kradumthong (KD), Monthong (MT), and Puangmanee (PM) to investigate genetic diversities of cultivated durians. KD, MT, and PM genome assemblies were 832.7, 762.6, and 821.6 Mb, and their annotations covered 95.7, 92.4, and 92.7% of the embryophyta core proteins, respectively. We constructed the draft durian pangenome and analyzed comparative genomes with related species in Malvales. Long terminal repeat (LTR) sequences and protein families in durian genomes had slower evolution rates than that in cotton genomes. However, protein families with transcriptional regulation function and protein phosphorylation function involved in abiotic and biotic stress responses appeared to evolve faster in durians. The analyses of phylogenetic relationships, copy number variations (CNVs), and presence/absence variations (PAVs) suggested that the genome evolution of Thai durians was different from that of the Malaysian durian, Musang King (MK). Among the three newly sequenced genomes, the PAV and CNV profiles of disease resistance genes and the expressions of methylesterase inhibitor domain containing genes involved in flowering and fruit maturation in MT were different from those in KD and PM. These genome assemblies and their analyses provide valuable resources to gain a better understanding of the genetic diversity of cultivated durians, which may be useful for the future development of new durian cultivars.

## 1 Introduction

*Durio zibethinus* (family Malvaceae) or durian is an endemic plant species of Southeast Asia (Teh et al., 2017). Cultivated durians produce thorny fruits, known as the “King of Fruit”, with sweet, delicious, and richly aromatic arils inside (Ketsa et al., 2020). The expensive price of their fruits makes durians an important economic crop in Thailand, Malaysia, and Indonesia (Ketsa et al., 2020). Several durian cultivars have been developed, *via* outcrossing and selection, and cultivated in these countries (Ketsa et al., 2020). Some popular durian cultivars in Thailand are Monthong, Kanyao, Kradumthong, Puangmanee, and Chanee. These cultivars exhibit different tree sizes, fruiting periods each year, fruit sizes, fruit ripening, and the number, flavor, texture, and aroma intensity of arils (Ketsa et al., 2020). The resistance levels to *Phytophthora* and other diseases also vary among cultivars (O’Gara et al., 2004; Ketsa et al., 2020). To understand the genetic basis of durian, the genome of Musang King cultivar has been sequenced (Teh et al., 2017). The analysis of this genome data provided insights into the genome evolution of *D. zibethinus*, including the expansion of the methionine ɣ-lyase gene family, which played a role in the production of volatile sulfur compounds (Teh et al., 2017). The reanalysis of this genome showed a hexaploidization event in the durian genome about 19–21 million years ago (Mya) (Wang et al., 2019). Another analysis of the same genome sequence revealed a catalog of 2,586 resistance gene analogs in this durian cultivar (Cortaga et al., 2022). This genome sequence has proven itself to be an important resource for understanding the agronomic characteristics and evolution of durian.

Plant genomes are dynamic and a single reference genome sequence for each species might not reflect genomic diversity within species (Danilevicz et al., 2020). The resequencing of genomes from multiple individuals with different phenotypes within a species and the construction of pangenome enabled the listing of total core genes (found in all individuals) and dispensable or accessory genes (found in subsets of individuals) (Bayer et al., 2020; Danilevicz et al., 2020; Della Coletta et al., 2021). One of the approaches for constructing a pangenome was map-to-pan, which included the mapping of resequenced reads to a reference genome, *de novo* assembly of unmapped reads, and the incorporating of assembled contigs with the reference genome (Hu et al., 2020; Della Coletta et al., 2021). Pangenomes were constructed from hundreds to thousands of individuals in sunflower (Hübner et al., 2019), tomato (Gao et al., 2019), and Asian rice (Wang et al., 2018). A much smaller number of individuals were used to construct the pangenomes of walnut (Trouern-Trend et al., 2020), banana (Rijzaani et al., 2022), maize (Haberer et al., 2020), and pepper (Kim et al., 2021). The key knowledge obtained from the analyses of pangenomes was extensive structural variants (SVs), including presence-absence variation (PAV) and copy number variation (CNV) (Della Coletta et al., 2021). These variations were shown to be linked to agronomic traits and were useful for crop improvement (Danilevicz et al., 2020). For example, the analyses of the pangenome showed the presence of an allele in the *TomLoxC* promoter that contributed to desirable tomato flavor and the negative selection of disease-resistance genes during the domestication of tomatoes (Gao et al., 2019). The variations of several disease-resistance gene families were reported from the analyses of walnut (Trouern-Trend et al., 2020) and pepper (Kim et al., 2021) pangenomes. The effects of SVs on agronomically important traits highlighted the value of genome resequencing on multiple individuals with different phenotypes within a species (Della Coletta et al., 2021).

In this study, we resequenced the genomes of three popular durian cultivars, including Kradumthong (KD), Monthong (MT), and Puangmanee (PM). The resequencing allowed us to identify the genetic variations among durian cultivars and between durians and other species. We also analyzed transcriptome data to see groups of transcripts highly expressed in different durian cultivars. The results improved our understanding of the durian genome evolutions and provided other genetic information that could be used to guide the breeding of new durian cultivars.

## 2 Materials and methods

### 2.1 Plant materials and DNA/RNA isolation

The MT, KD, and PM trees whose materials were used in this study were maintained at Chanthaburi horticultural research center, Chanthaburi, Thailand. They grow in the non-flooded area and receive 150 liters/tree/day of water supply. Fertilizers and pesticides are supplied regularly. For this study, healthy leaves were collected, immediately frozen in liquid nitrogen, and stored at −80°C. DNA was extracted and purified using the QIAGEN Genomic-tip 100/G following the manufacturer’s protocol (Qiagen, Germany). DNA quality was assessed using 0.75% pulsed-field gel electrophoresis and the concentration was tested with Qubit^®^ dsDNA BR Assay Kits (Thermo Fisher Scientific) and NanoDrop (Thermo Fisher Scientific).

Total RNA was extracted from healthy leaves using CTAB buffer and 25:24:1 phenol:chloroform:isoamyl alcohol. Contaminated DNA was removed by using DNA-free™ DNA Removal Kit (Invitrogen™). The quality and quantity of RNA were evaluated with the fragment analyzer machine (Agilent). Poly(A) mRNAs were enriched from total RNA samples using the Dynabeads mRNA Purification Kit (Thermo Fisher Scientific, Waltham, MA, USA).

### 2.2 Library preparation and sequencing

For library preparation and sequencing, one nanogram of high quality, high molecular weight DNA was used for the 10x Genomics linked-read library preparation using the Chromium Genome Library Kit & Gel Bead Kit v2, the Chromium Genome Chip Kit v2, and the Chromium i7 Multiplex Kit according to the manufacturer’s instructions (10x Genomics, Pleasanton, USA). The library quality was assessed using Bioanalyzer DNA High Sensitivity DNA Assay (Agilent) and the concentration was tested with Qubit^®^ dsDNA BR Assay Kits (Thermo Fisher Scientific). The 10x Genomics library was sequenced on the Illumina HiSeq X Ten (150 bp paired-end reads). For RNA, we constructed cDNA libraries according to the MGIEasy RNA Library Prep set protocol. The libraries were sequenced with the MGISEQ-2000RS machine.

### 2.3 Genome assembly

The linked-read data were assembled using the Supernova assembler version 2.1.1 with the default parameter setting (https://support.10xgenomics.com/de-novo-assembly/software/pipelines/latest/using/running; 10x Genomics, Pleasanton, USA). For the quality assessment, short-read DNA sequence data obtained from this study were mapped back to the final assembly sequences using minimap2 (Li, 2018), and the percentage of successful mapping was identified. We also employed the Benchmarking Universal Single-Copy Orthologs (BUSCO) version 4.0.5 (Simão et al., 2015) to evaluate the assembly by testing for the presence and completeness of the orthologs using the embryophyta OrthoDB release 10 database (Zdobnov et al., 2021).

### 2.4 Genome annotation

We used RepeatModeler (Flynn et al., 2020) with default parameters to construct libraries of the consensus sequences of TEs from the assemblies of four durian cultivars. The assembly of MK was retrieved from the Genomes – NCBI Datasets database. The consensus sequences of all cultivars provided by RepeatModeler were used with RepeatMasker version 4.1.2 (http://www.repeatmasker.org) with default parameters to identify repeats in all durian assemblies. The LTR compositions in each assembly were identified by LTRharvest (Ellinghaus et al., 2008) and LTR_FINDER_parallel (Ou and Jiang, 2019) with default parameters. The LTR annotations from both programs were used with LTR_retriever (Ou and Jiang, 2018) to calculate the insertion of times of LTR sequences based on the rate of nucleotide substitution per site per year of 3.5 × 10^−9^ (Wang et al., 2021).

We used MAKER2 (Holt and Yandell, 2011) to annotate gene regions in repeat masked sequences of MT, KD, and PM assemblies. The assembled transcript sequences of each durian cultivar and the protein sequences of Arabidopsis, grape, rice, and soybean were as described in the MK annotation (Teh et al., 2017). In the MAKER pipeline, this evidence was used to generate an initial set of gene predictions. Snap (Korf, 2004) and Augustus (Stanke et al., 2006) were used for ab initio gene predictions based on the first round of gene predictions. We employed the BUSCO version 4.0.5 (Simão et al., 2015) to evaluate the annotation results by testing for the presence and completeness of the orthologs using the embryophyta OrthoDB release 10 database (Zdobnov et al., 2021).

### 2.5 Comparative genomics and phylogenetic analysis

We used OrthoFinder (Emms and Kelly, 2019) to identify orthologous groups (protein families) from protein sequences of four durian cultivars (*Durio zibethinus*), two cottons (*Gossypium arboreum* and *Gossypium raimondii*) and cacao (*Theobroma cacao*), Arabidopsis (*Arabidopsis thaliana*) and papaya (*Carica papaya*). The protein sequences of Musang King durian, *G. arboreum*, *G. raimondii*, *T. cacao*, *A. thaliana*, and *C. papaya* were downloaded from the NCBI database with the accession numbers GCF_002303985.1, GCF_000612285.1, GCF_000327365.2, GCF_000208745.1, GCF_000001735.4, and GCF_000150535.2, respectively. The sequences from single-copy orthologous groups were aligned with MUSCLE software (Edgar, 2004; Edgar, 2021; https://github.com/rcedgar/muscle/). The alignments were further processed by trimming gap-rich regions with trimAl (Capella-Gutiérrez et al., 2009) using the automated1 heuristic method and concatenating with catsequences (https://github.com/ChrisCreevey/catsequences). The final concatenated alignment was subjected to ModelTest-NG (Darriba et al., 2020) for identifying the substitution model of each alignment block. The RAxML-ng (Kozlov et al., 2019; https://github.com/amkozlov/raxml-ng) with default MRE-based bootstrapping parameter was used to construct a maximum likelihood phylogenetic tree from the concatenated alignment and substitution models. Protein family expansions/contractions were analyzed with CAFE version 5 (Mendes et al., 2020) based on the numbers of proteins in each family and phylogenetic tree. Possible functions of protein families were annotated based on Gene Ontology (GO) and MapMan4 function classes. We performed sequence homology searches between the representative sequence (the longest protein sequence) of each family with the MapMan4 bins (Schwacke et al., 2019) using Mercator4 version 5.0 (https://plabipd.de/portal/mercator4) and the NCBI non-redundant protein sequences (nr) databases (https://ftp.ncbi.nlm.nih.gov/blast/db/) using Blast2GO (Conesa et al., 2005).

### 2.6 Presence/absence variation analysis

We used the EUPAN pipeline (Hu et al., 2017) to identify PAV profiles from annotated gene contents of the genomes. The following steps were adopted for each of the MT, KD, and PM *de novo* assemblies. The contigs were aligned to MK reference assemblies (Teh et al., 2017). The unaligned sequences were blasted against the NCBI nonredundant nucleotide (nr/nt) database (https://ftp.ncbi.nlm.nih.gov/blast/db/) to filter out contaminated sequences (we kept only sequences matched with plant sequences in the database) and redundant sequences were removed. The draft pangenome was built by combining the reference genome and a set of non-redundant sequences. We used Liftoff (Shumate and Salzberg, 2021) for the annotation by transferring the annotations of the original assemblies to the draft pangenome based on sequence identity of 90% and gene coverage of 80%. High-quality DNA reads, which were used for genome assemblies, were mapped to draft pangenome. Gene coverage and gene PAVs were calculated based on mapping results and the annotations.

For transcriptome analysis, quality RNA sequencing reads were aligned to the draft pangenome with HISAT2 and were assembled with StringTie to get full-length transcripts (Pertea et al., 2016). We followed the get_homologues-est pipeline (Contreras-Moreira et al., 2017) to process transcripts and to get PAV profiles of durian cultivars. In brief, coding regions of transcripts were obtained using transcripts2cdsCPP.pl, and clusters of orthologous sequences were generated with get_homologues-est.pl, PAVs were calculated with compare_clusters.pl and parse_pangenome_matrix.pl, and domain enrichment was obtained by using pfam_enrich.pl script. The assembled transcripts were annotated with GO and MapMan4 function classes as mentioned above.

## 3 Results

### 3.1 Genome assembly and annotation

We obtained 128.92, 119.96, and 115.08 Gb of raw reads from the genome sequencing of KD, MT, and PM cultivars, respectively. The raw reads represented 140-157X coverage of the 738 Mb of Musang King (MK) reference assembly (Teh et al., 2017). Using 10x Genomics linked-reads library sequencing and ragtag for reference-guided scaffolding, we obtained the assemblies of 839.7 (N50 = 21.6 Mb), 762.6 (N50 = 18.3 Mb), and 821.6 (N50 = 19.0 Mb) Mb for KD, MT and PM (**Table 1**), respectively. The numbers of scaffolds longer than 10 Mb (30 scaffolds) were the same among the three cultivars and MK (Teh et al., 2017). The alignments of KD, MT, and PM assembly sequences to the MK reference sequence showed contiguous matches of these scaffolds (**Supplementary Figure 1**). A total of 93.5%, 92.6%, and 91.3% of the KD, MT, and PM assemblies, respectively, could be aligned to the MK assembly (Teh et al., 2017). The results showed that 79.8%, 79.4%, and 76.8% of the KD, MT, and PM assemblies were aligned to the MK assembly with an identity value of >50%. The low percent identity areas were found in the alignment with the repeat regions of the MK assembly. These results revealed the variations of genome sequences among these cultivars. The numbers of annotated protein-coding genes in KD, MT, and PM assemblies were 47,980, 45,705, and 44,814, respectively (**Table 2**), which were similar to that in MK assembly (45,335) (Teh et al., 2017). BUSCO analyses of the annotations of KD, MT, and PM assemblies showed 95.7, 92.4, and 92.7% completeness based on the odb10 embryophyta database.

**Table 1:** Genome statistics.

	Kradumthong	Puangmanee	Monthong
Raw read (Gb)	128.92	115.08	119.96
N50 (bp)	21,597,206	19,022,827	18,254,889
Total bases (Mb)	839.66	821.59	762.61
Number of Contigs	59,633	99,407	92,437
Number of Contigs >10 Mb	30	30	30
Longest contig (bases)	37,760,038	30,624,853	32,015,873
GC content (%)	33.76	33.21	33.06
Repeat (%)	62.27	60.27	61.27
Gypsy LTR (%)	33.30	30.56	30.29

**Table 2:** Annotation statistics.

	Kradumthong	Puangmanee	Monthong
Number of predicted gene model	47,980	44,814	45,705
Total gene length (Mb)	136.68	132.40	130.25
Average gene size (nt)	2849	2954	2850
Average number of exons/genes	5.285	5.333	5.274
Average exon length (nt)	228.4	225.3	229.2
Average number of introns/genes	4.355	4.378	4.368
Average intron length (nt)	406.8	416.2	405.8
BUSCO (% of embryophyta core genes)	95.7	92.7	92.4

The unaligned fragments of MT, KD, and PM assemblies were merged with the MK reference assembly to generate the draft pangenome of these four popular durian cultivars (Hu et al., 2017). Only sequences that were equal to or longer than 1 kb were kept for downstream analyses. The total length of this draft pangenome was 745.9 Mb (31 Mb sequences were added to the MK reference assembly). The annotations of MK, KD, MT, and PM assemblies were transferred to the pangenome based on sequence alignments (Shumate and Salzberg, 2021). KD, MT, and PM annotations mapped to the same regions as MK annotations were filtered out. A total of 77,401 annotated proteins (from 50,112 genes) were transferred to the draft pangenome. The transferred proteins included all 63,007 annotated proteins from the MK assembly and 4,994, 3,074, and 6,326 proteins from KD, MT, and PM assemblies, respectively.

### 3.2 The analyses of repeat regions

We obtained about 1,200 consensus sequences of repeats in MK and KD (with a mean length of 1,471 and 1,534 bases, respectively) and about 1,600 sequences in MT and PM (with a mean length of 1,031 and 1,049 bases, respectively). About 60-63% (451-518 Mb) of these assemblies were masked as repeat regions. Among all repeats, the Gypsy elements of the long terminal repeat retrotransposon class (LTR/Gypsy) occupied the largest proportion of the assemblies (30-33%). We identified LTR/Gypsy elements in all four durians, cacao (*Theobroma cacao*), and two cotton species (*Gossypium arboreum*, *Gossypium raimondii*) for the interspecies comparative analysis. The numbers of intact LTR/Gypsy in MK and KD (228 and 105 sequences) were higher than that in MT and PM (30 and 23 sequences) and cacao (88 sequences). LTR/Gypsy elements in all durians were present in a significantly lower number than those in cottons (591 sequences in *G. raimondii* and 2,402 sequences in *G. arboretum*). The insertion times of LTR/Gypsy elements (the first appearance time of these elements in genomes) were estimated to understand their evolution in genomes. The average insertion times of all LTR/Gypsy elements in MK, KD, MT, and PM were estimated to be 7.6 ± 4.7, 10.1 ± 4.7, 11.8 ± 3.8, and 11.0 ± 4.9 Mya, respectively. The alignment and phylogenetic analysis of the LTR/Gypsy elements showed that the insertion time of the elements that could be found only in durians was estimated to be 8.2 ± 2.8 Mya. We found one LTR/Gypsy group in the phylogenetic tree that contained the elements from cacao (1 sequence), durians (25 sequences), and cottons (22 sequences) assemblies, and their insertion times were 21.9, 13.4 ± 3.7, and 5.0 ± 3.8 Mya, respectively (**Supplementary Figure 2**). The results together suggested that the amplification of LTR/Gypsy elements was most active in cottons. Among durian cultivars, the amplification was most active in MK.

### 3.3 Comparative analysis

**Figure 1:**
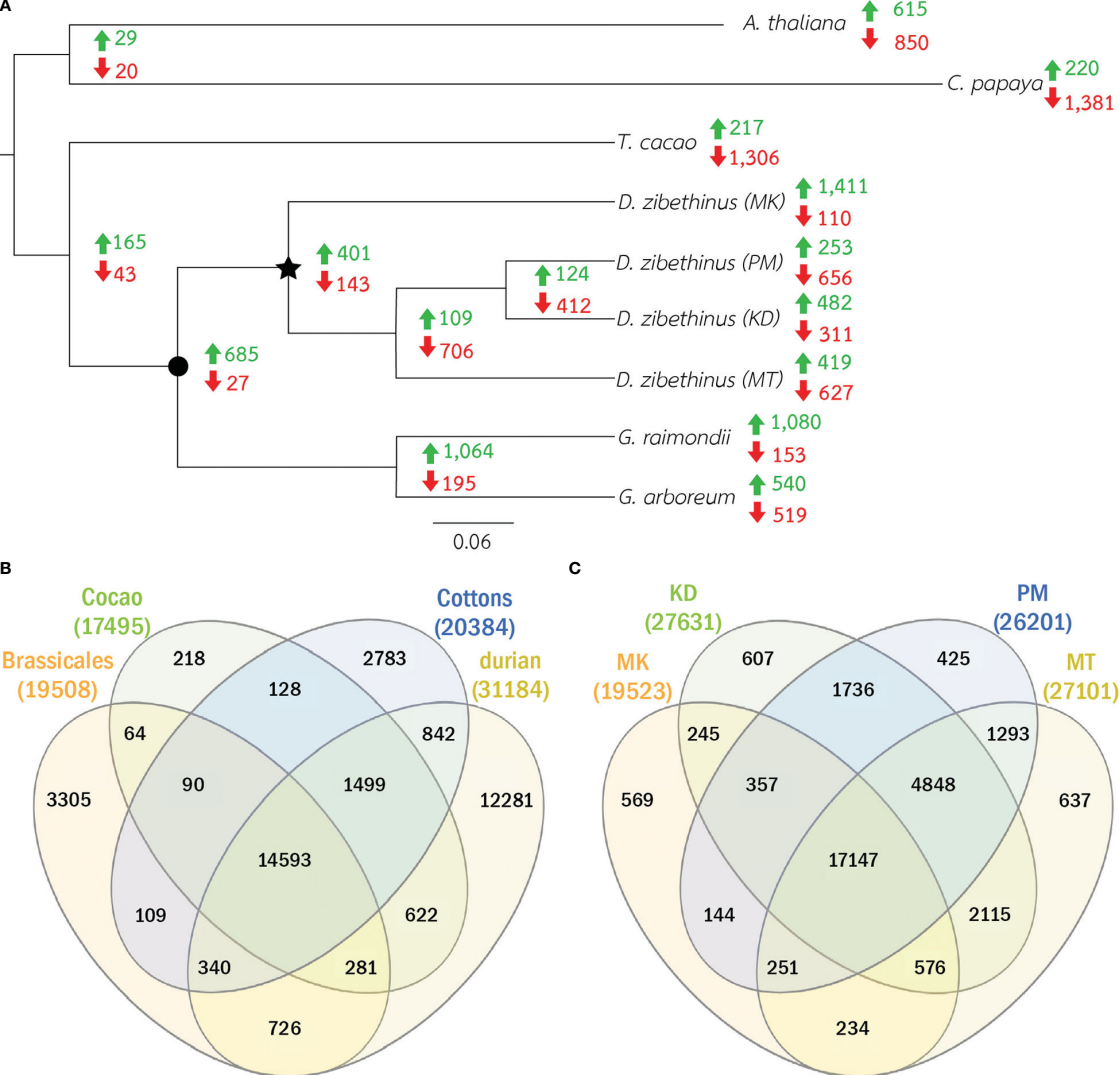
Phylogenetic tree and the numbers protein families. **(A)** The phylogenetic relations of four durian cultivars and other five related species are shown. Star node represents common ancestor of all durians and circle node represents the common ancestor of cottons and durians. Green and red numbers at nodes and leaves showed the numbers of expanded and contracted families. **(B)** The Venn diagram of the numbers of protein families in durians, cottons, cacao and Brassicales species (Arabidopsis and papaya) is shown. **(C)** The Venn diagram of the numbers of protein families in all four durian cultivars is shown.

We identified protein families by comparing the protein sequences of all durians with those of cottons (*G. arboretum* and *G. raimondii*), cacao (*T. cacao*), Arabidopsis (*Arabidopsis thaliana*) and papaya (*Carica papaya*). Cottons and cacao were representatives of Malvales species, while Arabidopsis and papaya were Brassicales species and were considered outgroups. We built a maximum likelihood phylogenetic tree from the sequences in 314 single-copy protein families. The tree showed that durians and cottons formed a monophyletic clade that was split from cacao (**Figure 1A**). Within the durian group, MK was isolated from other durians and, for the group of durians of Thailand, MT was separated from PM and KD.

**Figure 2:**
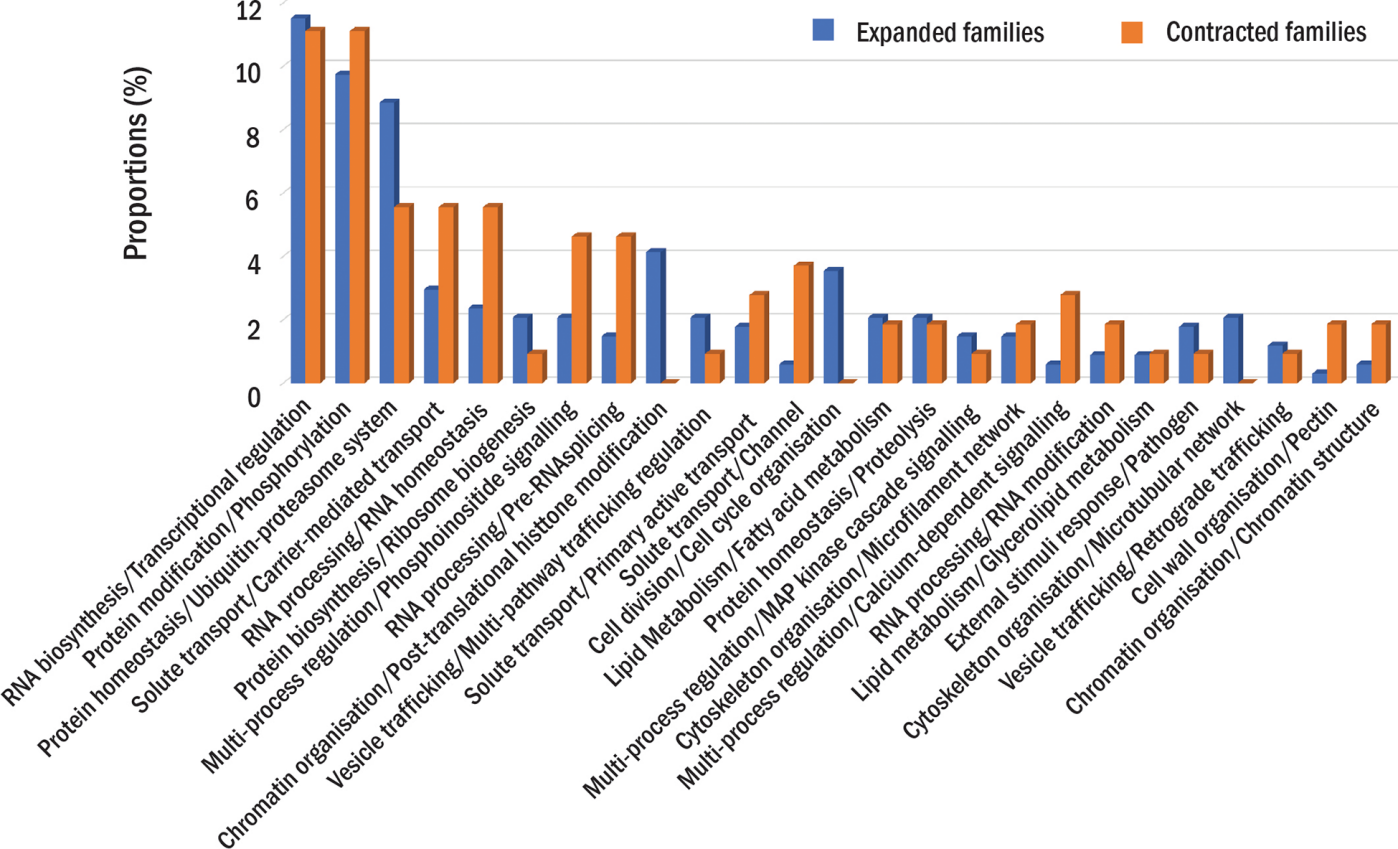
Protein families in the durian ancestor. The proportions of expanded (blue) or contracted (orange) families in each function class are shown. The proportions are calculated as the percentage of the number expanded or contracted families in each function class to the total number of expanded or contracted families.

The comparative analysis showed that 31,184 protein families had proteins from at least one durian cultivar (**Figure 1B**). Comparison among durian cultivars showed that about 1-2% of the families with durian proteins were cultivar specific (**Figure 1C**). Although both durian and cotton were in the clade that split from cacao (**Figure 1A**), the number of durian-cacao specific families (the families commonly found only in durians and cacao, 622 families) was about five times larger than that of cotton-cacao specific families (128 families). These results suggested that the rates of protein evolution in durian and cotton were different. We identified expanded and contracted families for every internal and leaf node within the phylogenetic tree to see the evolution of protein families (**Figure 1A** and **Supplementary Table 1**). The analysis revealed 401 expanded and 143 contracted families in the durian common ancestor (**Figure 1A**) when compared to the families in the common ancestor of cotton and durian (**Figure 1A**). Functions of protein families were annotated and classified based on MapMan function classes (Schwacke et al., 2019). The results showed that the families of proteins involved in cell division were expanded in the durian common ancestor. We found high proportions of both expanded and contracted families involved in transcriptional regulation and protein phosphorylation (**Figure 2**). The proportion of expanded families was higher than the number of contracted families for the proteins involved in, for example, ribosome biogenesis and pathogen response. On the other hand, the proportion of contracted families was higher than that of the expanded families for the proteins involved in solute transport and pectin metabolism.

**Figure 3:**
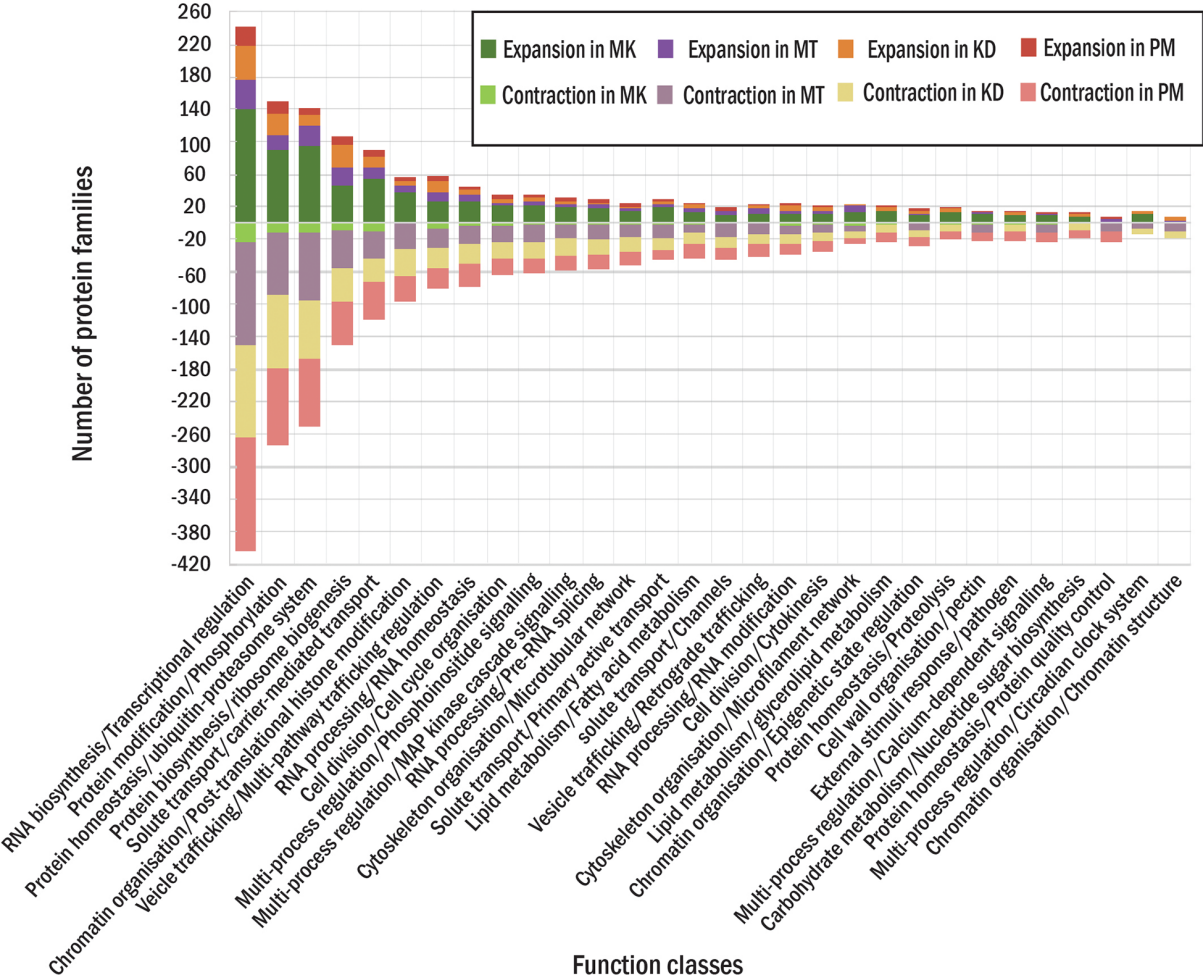
Protein families in each durian cultivars. The numbers of expanded (positive side) and contracted (negative side) families in each function class for MK, MT, KD and PM are shown in different colors.

For four durian leaf nodes, the analysis revealed the expansion of 1,617 protein families (the families found in multiple durian cultivars were counted only once) and the contraction of 1,330 families (**Figure 1A**). We also classified these families based on MapMan function classes and found several classes that contained both contracted and expanded families from the same cultivar. We selected the top 30 function classes with the highest difference between the numbers of expanded and contracted families to see highly adaptive protein functions in durians (**Figure 3**). Like those of the durian common ancestor, most of the rapidly evolved protein families in each durian cultivar were involved in transcriptional regulation and protein phosphorylation (**Figure 3**). These proteins were also involved in the responses of plants to abiotic and biotic stresses, the sensing of light quality, and the regulation of plant growth and organ development. The number of protein members of these two and most of the other families were the highest in MK and declined in KD, MT, and PM, respectively. The adaptability of some families was lesser in some durian cultivars than that in other cultivars. For example, the families of proteins related to pathogen response were present in the list of the top 30 most adaptive functions of all cultivars but not in the list of MT, indicating that these families were less adaptive in MT. The families involved in pectin metabolism and the circadian clock system were less adaptive in KD and PM, respectively. Some other families were highly adaptive in only one cultivar. The examples were families involved in nucleus protein translocation in MK, light response in MT, s-glutathionylation protein modification in KD, and sucrose metabolism in PM.

### 3.4 Presence/absence variations

We analyzed the gene presence-absence variations (PAVs) among cultivars based on the annotation of the draft pangenome. The analysis showed that 49,631 genes or about 99% of the total genes were present in all analyzed cultivars (**Supplementary Figure 3**). We found 414 genes that were absent in one to three cultivars. These genes were referred to as PAV genes. To reduce redundancy, sequences with 40% identity and 30% coverage to their longer homologous sequences were filtered out. The putative functions of PAV genes were annotated based on the homology search against the NCBI database. The PAV sequences matched with the transposon-related functions and uncharacterized genes were removed. We searched for the homologs of the PAV genes in the protein family analysis results. The PAV genes that had homologs in the same genome were discarded. Some PAV genes were not assigned to any protein family due to the uniqueness of their sequences. This group of PAV genes was kept for further analysis.

**Figure 4:**
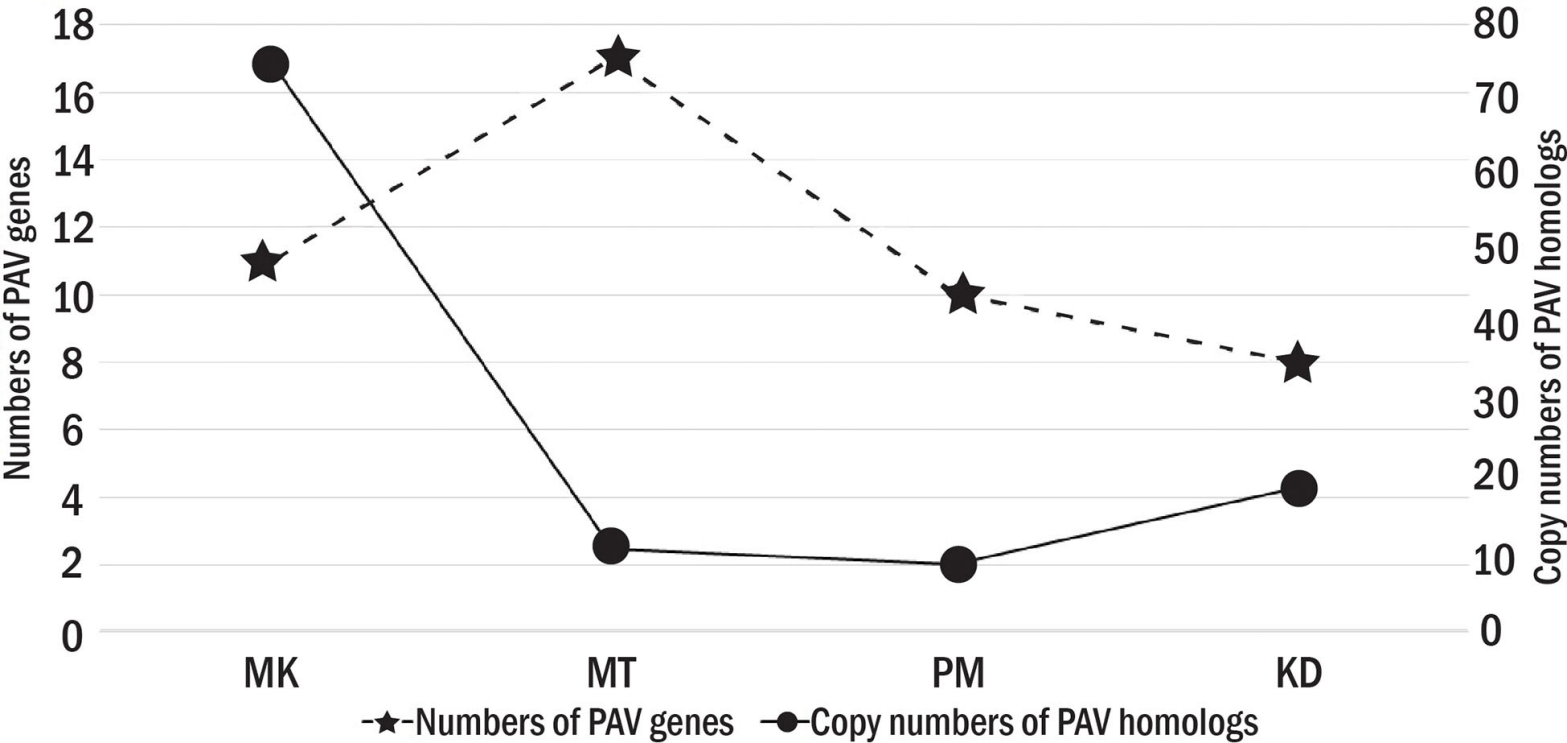
The number of PAV genes and PAV homologs. The primary y axis shows the numbers of PAV genes with star symbols that are connected by dash lines. The secondary y axis shows the copy numbers of PAV homologs with circle symbols that are connected by solid lines.

We finally obtained 39 PAV genes (**Table 3**). Among them, seven PAV genes were simultaneously absent in more than one cultivar, while other 32 PAV genes were absent in a cultivar-specific manner. The numbers of PAV genes that were specifically absent in MK, MT, KD, and PM were nine, eleven, five, and seven, respectively (**Figure 4**). For each cultivar, the homologs of each of its PAV genes were searched in the genomes of the other three cultivars based on the protein family analysis results. These homologs were referred to as PAV homologs. We counted the copy number of PAV homologs and found that the PAV genes in MK had the highest number of homologs (**Figure 4**). For example, the cytochrome P450 78A7-like encoding gene was the PAV gene in MK and the copy numbers of the homolog of this gene in KD, PM, and MT were twenty-one, five, and two, respectively (**Table 3**). Other PAV genes in MK with similar PAV homolog profiles were UBN2 domain-containing protein and microtubule-associated protein TORTIFOLIA coding genes. The total copy number of the PAV homologs in MK (75 copies) was significantly higher than those in MT, KD, or PM (**Figure 4**). These results suggested that the MK genome might evolve differentially from the MT, KD, and PM genomes.

**Table 3:** List of selected PAV genes and the numbers of proteins in the associated protein families.

	PAVs*				Protein Numbers			
Annotations	MK	MT	KD	PM	MK	MT	KD	PM
cytochrome P450 78A7-like	0	1	1	1	0	2	21	5
cytochrome P450 CYP736A12-like	0	1	1	1	0	0	1	1
MDIS1-interacting receptor like kinase 2-like	0	1	1	1	0	0	0	2
microtubule-associated protein TORTIFOLIA1 isoform X1	0	1	1	1	0	1	2	4
non-functional NADPH-dependent codeinone reductase 2-like	0	1	1	1	0	2	5	1
probable disease resistance protein At1g12280	0	1	1	1	–	–	–	–
putative sel repeat-containing protein L21	0	1	1	1	0	0	1	2
putative wall-associated receptor kinase-like 16	0	1	1	1	0	1	1	1
UBN2 domain-containing protein	0	1	1	1	0	6	9	5
lysine histidine transporter 1	1	0	1	1	–	–	–	–
NADH-plastoquinone oxidoreductase subunit 7	1	0	1	1	–	–	–	–
NB-ARC domain-containing disease resistance protein, putative	1	0	1	1	1	0	0	0
octapeptide-repeat protein T2-like	1	0	1	1	–	–	–	–
PLAC8 family protein	1	0	1	1	–	–	–	–
probable disease resistance protein At1g12280 isoform X2	1	0	1	1	3	0	0	0
putative F-box only protein 10	1	0	1	1	–	–	–	–
receptor-like protein 12	1	0	1	1	–	–	–	–
receptor-like protein 9DC3 isoform X2	1	0	1	1	–	–	–	–
serine/threonine-protein kinase PBL13 isoform X1	1	0	1	1	–	–	–	–
SKP1-like protein 1A	1	0	1	1	–	–	–	–
cycloartenol-C-24-methyltransferase	1	1	0	1	0	1	0	1
F3H9.20 protein	1	1	0	1	1	1	0	0
glutamate dehydrogenase 1	1	1	0	1	–	–	–	–
glycine-rich cell wall structural protein 1-like	1	1	0	1	4	4	0	4
G-type lectin S-receptor-like serine/threonine-protein kinase At4g27290	1	1	0	1	–	–	–	–
arginase 1, mitochondrial	1	1	1	0	–	–	–	–
cytochrome P450 81D1-like	1	1	1	0	1	1	0	0
cytosolic sulfotransferase 15-like	1	1	1	0	1	0	1	0
transcription factor MYB16-like	1	1	1	0	–	–	–	–
tryptophan synthase beta chain 1	1	1	1	0	–	–	–	–
tyrosine-protein phosphatase RLPH2	1	1	1	0	–	–	–	–
zinc finger BED domain-containing protein RICESLEEPER 2-like	1	1	1	0	2	0	1	0
beta-glucuronosyltransferase GlcAT14A-like isoform X1	0	0	1	1	–	–	–	–
serpin-ZX-like	0	0	1	1	0	0	1	1
putative DNA (cytosine-5)-methyltransferase CMT1	1	0	0	1	1	0	0	0
zinc finger CCCH domain-containing protein 1-like	1	0	0	1	1	0	0	1
glycoside hydrolase family 2 protein	1	0	1	0	–	–	–	–
Histone acetyltransferase MCC1	1	0	1	0	1	0	1	0
glutamate dehydrogenase 1	1	1	0	0	–	–	–	–

*0 = presence, 1 = absence

The number and function of the PAV genes also varied among Thai durian cultivars (**Figure 4** and **Table 3**). The total number of PAV genes was highest in MT, followed by those in PM and KD, respectively. Despite their high number, the PAV genes in MT had a low copy number of PAV homologs (**Figure 4**). Additionally, several genes missing in MT were potentially involved in defense response. Examples of these PAV genes were probable disease resistance protein At1g61300, probable disease resistance protein At1g12280, NB-ARC domain-containing disease resistance protein, and lysine histidine transporter 1 encoding genes (**Table 3**). The results indicated that the PAV genes in MT were present as accessory genes in other cultivars. For PM and KD, the numbers of PAV genes in these two cultivars were similar and were lower than that in MT (**Figure 4**). The numbers of their PAV homologs appeared to be lower than that in MK. Additionally, most of the genes absent in KD and PM were involved in the metabolism of biomolecules, which differed from the functions of genes absent in MT (**Table 3**). These results together showed three different PAV profiles in MK, MT, and the group of KD and PM, which was related to the phylogenetic relationship and origins of these durian cultivars.

### 3.5 Pantranscriptome

**Figure 5:**
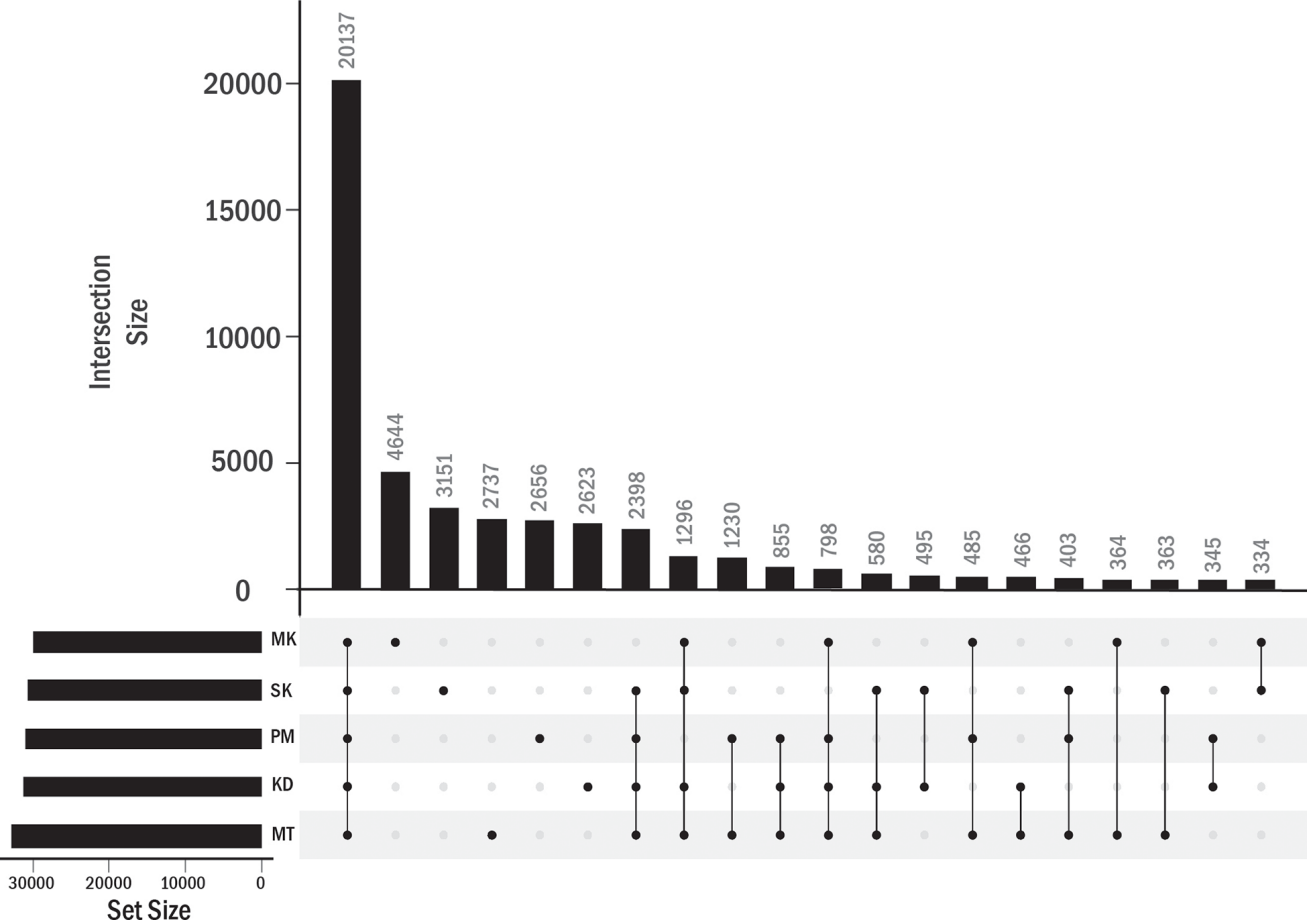
Transcript orthologous groups in each durian cultivars. The number of orthologous groups in each durian cultivars is shown by the horizontal bar. Vertical bars showed the numbers of orthologous groups found in single (single dot in column) cultivar or shared by multiple cultivars (connected dots).

We identified the PAVs at the gene expression level. In addition to KD, MT, PM, and MK durians, Salika (SK) cultivar was included in this analysis. The leaf samples of KD, MT, PM, and SK were obtained from plants grown under the same environment in a small cultivation plot (the distance between plants was about 10 meters) at the Chanthaburi horticultural research center. MK was considered as an outgroup in this analysis as this cultivar grew under a different environment. The analysis of homology-based pantranscriptome analysis showed a total of 48,779 transcript orthologous groups. We found that 41.3% of these orthologous groups contained transcripts of all five cultivars (core orthologous groups). The domain enrichment analysis (based on the protein sequences translated from transcript sequences) using all orthologous groups as background showed the enrichment of helicase conserved C-terminal domain, pentatricopeptide repeat domain, and F-box domain in the core group (**Supplementary Table 2**). A majority of sequences with helicase conserved C-terminal domain were members of the DEAD-box ATP-dependent RNA helicase family. We found that 32.3% of all orthologous groups contained transcripts from single cultivars, including 9.5%, 6.4%, 5.6%, 5.4%, and 5.4% from MK, SK, MT, PM, and KD, respectively (**Figure 5**). No enriched domains were found for each of these groups.

The transcripts in the other 58.7% orthologous groups were not expressed in at least one cultivar (**Figure 5**). These orthologous groups were referred to as accessory orthologous groups. The number of accessory groups in MT (25.9% of the total orthologous groups) was higher than that in KD (22.8%), PM (22.1%), SK (21.4%), and MK (20.2%), respectively. Some domains could be enriched in multiple cultivars because some accessory groups were shared by multiple cultivars (**Table 4**). The domains enriched in the accessory groups of four cultivars included multicopper oxidase domain (unenriched in MK and enriched in all other cultivars), no apical meristem (NAM) protein (unenriched in PM), and late embryogenesis abundant protein (unenriched in SK) (**Supplementary Table 2**). Examples of enriched domains in three cultivars were the thaumatin family (thaumatin-like protein) in MT, KD, and PM, probable lipid transfer (xylogen-like protein) in MT, PM and SK, and SRF-type transcription factor (agamous-like MADS-box protein) in MK, PM, and SK. Some domains enriched in two cultivars were AP2 (ethylene-responsive transcription factor), xylanase inhibitor N-terminal (aspartyl protease family protein), gibberellin regulated protein (GAST1 protein homolog and Snakin-1), and pectinesterase (probable pectinesterase) domains in MT and PM, wall-associated receptor kinase galacturonan-binding (rust resistance kinase Lr10) domain in MT and KD, and NB-ARC (putative disease resistance families) domain in KD and PM. Finally, the domains enriched in single cultivar included, X8, peroxidase, plant invertase/pectin methylesterase inhibitor, C2H2-type zinc finger, and epidermal patterning factor protein domains in MT; d-mannose binding lectin, s-locus glycoprotein, and berberine like domains in MK; MYB-like DNA-binding domain in SK; and auxin-responsive protein, transcriptional repressor-ovate, cotton fiber expressed protein, and sulfotransferase domain in PM.

**Table 4:** List of selected enriched domains .

PfamID	Description	Enrichment*				
		KD	MT	MK	PM	SK
PF00394	Multicopper oxidase	1	1	0	1	1
PF02365	No apical meristem (NAM) protein	1	1	1	0	1
PF03168	Late embryogenesis abundant protein	1	1	1	1	0
PF00560	Leucine Rich Repeat	1	0	1	1	0
PF00314	Thaumatin family	1	1	0	1	0
PF02984	Cyclin, C-terminal domain	1	1	0	0	1
PF14368	Probable lipid transfer	0	1	0	1	1
PF00319	SRF-type transcription factor	0	0	1	1	1
PF13947	Wall-associated receptor kinase galacturonan-binding	1	1	0	0	0
PF00931	NB-ARC domain	1	0	0	1	0
PF00847	AP2 domain	0	1	0	1	0
PF14543	Xylanase inhibitor N-terminal	0	1	0	1	0
PF02704	Gibberellin regulated protein	0	1	0	1	0
PF00190	Cupin	0	1	0	1	0
PF03188	Eukaryotic cytochrome b561	0	1	0	1	0
PF05910	Plant protein of unknown function (DUF868)	0	1	0	1	0
PF01095	Pectinesterase	0	1	0	1	0
PF00786	P21-Rho-binding domain	0	1	0	1	0
PF00462	Glutaredoxin	0	1	0	1	0
PF07983	X8 domain	0	1	0	0	0
PF00141	Peroxidase	0	1	0	0	0
PF04043	Plant invertase/pectin methylesterase inhibitor	0	1	0	0	0
PF13912	C2H2-type zinc finger	0	1	0	0	0
PF17181	Epidermal patterning factor proteins	0	1	0	0	0
PF02519	Auxin responsive protein	0	0	0	1	0
PF04844	Transcriptional repressor, ovate	0	0	0	1	0
PF02469	Fasciclin domain	0	0	0	1	0
PF05553	Cotton fibre expressed protein	0	0	0	1	0
PF00332	Glycosyl hydrolases family 17	0	0	0	1	0
PF00685	Sulfotransferase domain	0	0	0	1	0
PF00249	Myb-like DNA-binding domain	0	0	0	0	1
PF01453	D-mannose binding lectin	0	0	1	0	0
PF00954	S-locus glycoprotein domain	0	0	1	0	0
PF08031	Berberine and berberine like	0	0	1	0	0

*0 = unenriched, 1 = enriched

## 4 Discussion

Durian exhibited high genetic diversities because they are generally highly outcrossed during cultivation (Teh et al., 2017; Mursyidin et al., 2022). In this study, we sequenced the genomes of three popular durian cultivars of Thailand (Kradumthong, Monthong, and Puangmanee) and comparatively analyzed them with the genome of the Malaysian durian (Musang King) and other related species. The aim was to identify the similarities and differences among durian genomes to understand their evolution and diversity.

Assembly sequences of MT, KD, and PM were aligned with MK reference sequences to identify their conserved sequences. The alignments showed 30 conserved scaffolds among four durian genomes. The chromosome numbers of *Durio* species varied between 2n = 54 and 69, and the chromosome number of *D. zibethinus* was 2n = 56 (IBPGR, 1986). The number of conserved scaffolds was close to the estimated haploid chromosome number. A set of nonredundant DNA fragments was collected using the map-to-pan strategy (Hu et al., 2017) to generate the draft pangenome of all four popular durian cultivars. Size of the draft pangenome was increased by only 4% compared to size of the MK reference assembly because genome sequences were highly similar among four durians. Correspondingly, in sense of the genome annotations, the protein family analysis showed that only 1-2% of the families were unique to each durian cultivar.

The evolution of durian genomes among Malvales species was investigated by comparing protein families and LTR/Gypsy repeat elements in durians with those in cottons and cacao. In the phylogenetic tree, durians and cottons were in a monophyletic clade that was split from cacao (**Figure 1A**). The insertion timelines of LTR/Gypsy sequences in these genomes were consistent with this placement, *i.e.*, the repeats were the oldest in cacao, followed by those in durians and cottons, respectively. We also found that the number of protein families that cacao specifically shared with durians was higher than that it specifically shared with cotton (**Figure 1B**). These results indicated that changes in durian proteins after the split from the cacao-durian ancestor were lower than those in cotton proteins. The number of intact LTR/Gypsy sequences also suggested that the amplification of these elements was less active in durian genomes than that in cotton genomes. The results were consistence with the finding that the evolution rate of the durian genome was significantly slower than that of cotton genomes (Wang et al., 2019).

The expansion and contraction of protein families in the common ancestors of durians and cottons were compared to see the difference in their evolution paths. Several rapidly evolved families in durians were involved in transcription, protein phosphorylation, and protein ubiquitination processes. The rapid evolutions of these families have been linked to their functions, which were involved in the interactions of an organism with environments (Demuth and Hahn, 2009). For example, we found several expanded families of transcription factors (TF) that were involved in the responses of plants to pathogens and other environmental stimuli. These TFs included transcription factor MYC2 (*MYC2*) family, which could play roles in abiotic and biotic stress responses and the circadian clock (Kazan and Manners, 2013), probable WRKY transcription factor 53 family (*WRKY53*), which played roles in JA signaling, leaf senescence (Miao and Zentgraf, 2007) and the basal resistance against *Pseudomonas syringae* (Hu et al., 2012), and probable WRKY transcription factor 33 (*WRKY33*), which could confer resistance to fungal pathogens *Botrytis cinerea* and *Alternaria brassicicola* (Zheng et al., 2006). These families were expanded (*MYC2*), contracted (*WRKY33*), or unchanged (*WRKY53*) in the common ancestor of cotton. Other protein families related to pathogen responses and cell cycle were also highly expanded in durians. The results suggested that the evolutions of the proteins implicated in the responses of plants to environmental stimuli, especially pathogen infections, were different between durian and cotton. The expansion of these families might be related to the demand for a higher dosage of defense-responsive genes in durians to survive in high rainfall regions compared to cottons that were cultivated in arid to semiarid regions of the tropics and subtropics (Demuth and Hahn, 2009).

In this study, we found several similarities and differences among durian genomes. The phylogenetic tree and the profiles of CNV and PAV showed that MK was most different from all other cultivars (**Figures 1**, **3** and **Table 3**). Among the durians of Thailand, MT was isolated from KD and PM in the phylogenetic tree (**Figure 1A**). PAV and CNV analysis results showed that pathogen-responsive genes were one of the gene groups that exhibited high variations among durian cultivars. For example, we found a higher copy number of putative disease resistance RPP13-like protein 1, which conferred resistance to downy mildew caused by *Peronospora parasitica* (Bittner-Eddy et al., 2000), in MK than that in other cultivars. In contrast, the cysteine-rich receptor-like protein kinase 8 (*CRK8*) family, which could confer resistance to *P. syringae* in cottons (Hussain et al., 2022), was significantly contracted in MK but expand in KD and MT. Correspondingly, the resistance levels against pathogen infections have been shown to vary among durian cultivars (Vawdrey et al., 2005). The diversification of disease-resistance genes could be occurred not only after speciation but also after the divergence within species (Kim et al., 2021). Some redundant resistance genes might be deleted after whole-genome duplication events, while some other resistance genes might be retained related to the presence of particular pathogen pressures (Golicz et al., 2016; Rijzaani et al., 2022). The variation of gene contents in cottons could also be associated with geographic disjunction (Grover et al., 2017). For durian, MK was popularly grown in Malaysia, while MT was originally grown in the southern region of Thailand and PM and KD were originally grown in the central region of Thailand. The variation of gene contents among durian cultivars might be linked to the differences in their cultivation areas and breeding programs.

The expression of genes involved in flower formation and fruit ripening varied among durian cultivars. From the transcriptome analysis, we found the enrichment of plant invertase/pectin methylesterase inhibitor (PMEI) domain in MT. PMEI played a role in flower formation, fruit development, and biotic stress responses (Coculo and Lionetti, 2022). At the time that we collected samples, MT, KD, PM, and SK were in the flowering stage. Young fruit setting was also detected. The detection of transcripts with the PMEI domain might be related to flowering. The duration of fruit maturity varied among durian cultivars (Somsri, 2014). The growth and development period from anthesis to maturity of MT fruit was 120-127 days, which was longer than 95-100 days in KD (Sangwanangkul and Siriphanich, 2000; Somsri, 2014). Different maturity periods might be related to different levels of the transcript with the PMEI domain. Another gene whose expression was associated with fruit ripening in durian was the aminocyclopropane-1-carboxylic acid synthase encoding gene (*ACS*) (Teh et al., 2017). The expressions of *ACS* appeared to be similar among durian cultivars (Teh et al., 2017). In this study, the ACS protein family was shown to be contracted in papaya and cacao and expanded in cottons and durians, which was consistent with the study in MK (Teh et al., 2017). The copy numbers of this gene differed slightly among durian cultivars and cottons. In contrast to *ACS* that was a key ethylene-production enzyme in durian ripening (Liu et al., 2015; Teh et al., 2017), the expression of PMEI containing genes was not directly induced by ethylene (Srivastava et al., 2012). The role of the PMEI domain in durian maturity could be of interest to further research.

## 5 Conclusion

In this study, we resequenced the gnomes of three popular and agronomically different durian cultivars in Thailand (MT, KD, and PM) and comparatively analyzed them with the genomes of Malaysian durian (MK) and other related species to understand their genetic diversity. We found slower evolution rates of protein-coding genes and repeat elements in durian genomes compared to those in cotton genomes. Among durian cultivars, the highest expanded protein families in the Malaysian durian cultivar, which was different from Thai durian cultivars as shown in the phylogenetic tree. Among Thai durian cultivars, MT was most different from the other two Thai cultivars. The families of proteins involved in pathogen response in MT were less adaptive than those in KD and PM cultivars. We also observed that the number of homologs missing in MK but present in MT, KD, or PM genomes was higher than the number of homologs missing in MT, KD, or PM but present in the MK genome. The PAV analysis also showed that the missing genes in MT were involved in pathogen response, while the missing genes in KD and PM were involved in the metabolism of biomolecules. Additional analysis showed a higher abundance of transcripts with PMEI domain in MT than those in other cultivars, which was of interest to further test if the expression of the genes of these transcripts was involved in durian fruit ripening. Our results demonstrated genetic variations among the selected durian cultivars, which yielded arils with different flavors and textures and had different disease resistance levels. It was likely that these four durian cultivars were developed from different origins. In this study, we reported the draft version of the pangenome generated from the high-quality assemblies of four famous durian cultivars. For further works, commercial, local, and wild durian cultivars should be considered to construct a more complete version of the durian pangenome.

## Data availability statement

The datasets presented in this study can be found in online repositories. The genome sequences and RNA reads presented in the study are deposited in the NCBI database (https://www.ncbi.nlm.nih.gov/), accession number PRJNA641506 and PRJNA909034.

## Author contributions

WN performed bioinformatics analyses and wrote the manuscript. CN and SC performed bioinformatics analyses. SU-T and NN extracted DNA and RNA and performed sequencing. OC maintained durian plants and collected leaf samples. ST and WP initiated the project and revised the manuscript. All authors contributed to the article and approved the submitted version.

## Supplementary material

Supplementary Figure 1 Whole genome sequence alignments. **(A-C)** Dot pots of the alignments of **(A)** MT, **(B)** KD and **(C)** PM assembly sequences (on vertical axis) to the MK reference sequence (on horizontal axis) are shown. The alignment regions with the identity of >80% are in green color, while those with the identity of <80% are in orange color.

Supplementary Figure 2 The phylogenetic tree of LTR/Gypsy elements in the studied Malvales genomes. The LTR/Gypsy elements of cacao (CO), cottons (GA = *G. arboreum* and GR = *G. raimondii*) and durians are shown in orange, pink and blue colors.

Supplementary Figure 3 The numbers of DNA mapped genes. The Venn diagram showed the numbers of genes on the draft pangenome that are mapped by DNA reads of each durian cultivar.
