# Supplementary figures and images for: Resequencing of durian genomes reveals large genetic variations among different cultivars
Source: Front Plant Sci. 2023 Feb 16;14:1137077. doi: 10.3389/fpls.2023.1137077 (PMC9978785; doi:10.3389/fpls.2023.1137077)

# B

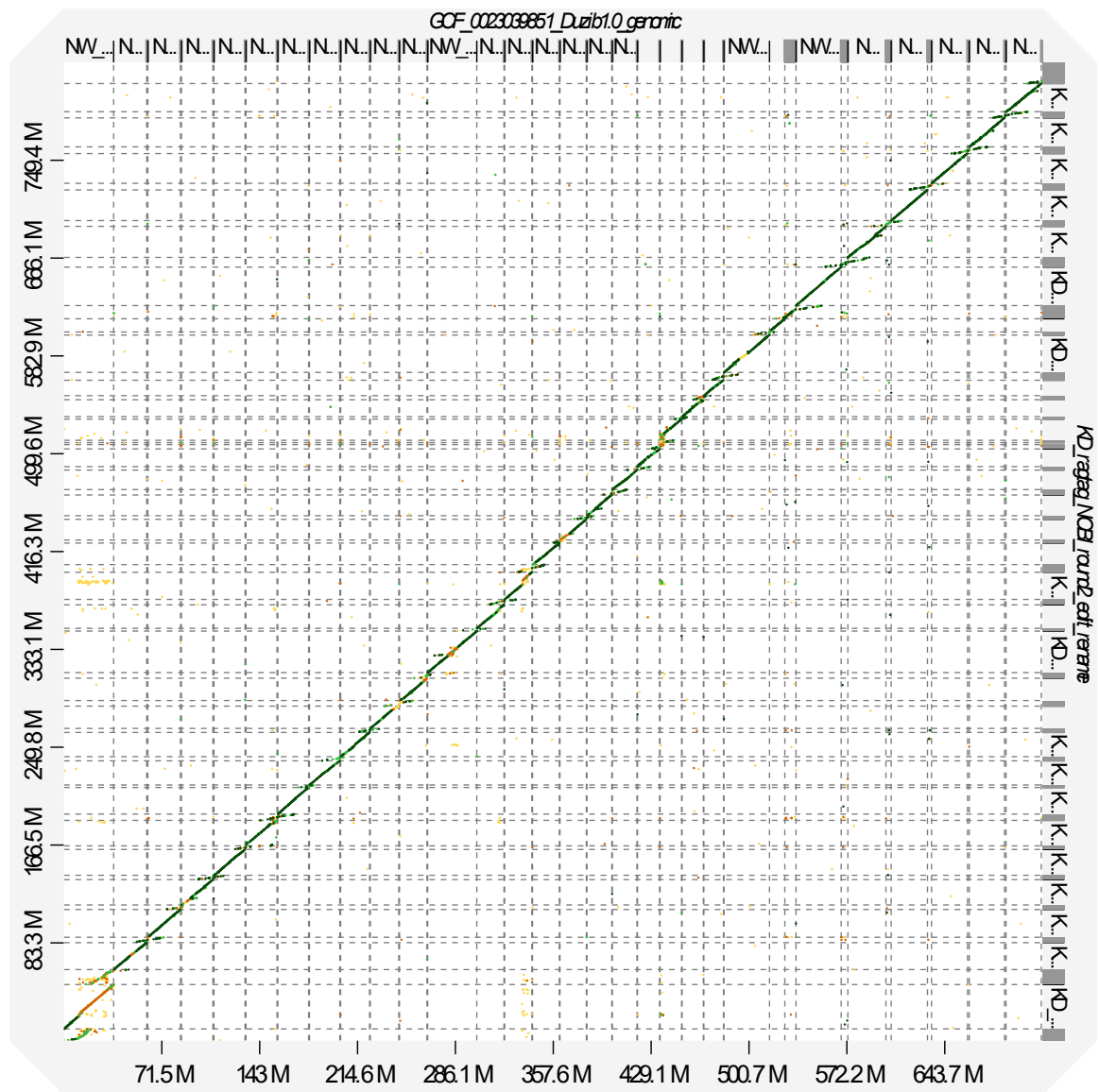

Supplement: Supplementary Figure 1 — Whole genome sequence alignments. (A-C) Dot pots of the alignments of (A) MT, (B) KD and (C) PM assembly sequences (on vertical axis) to the MK reference sequence (on horizontal axis) are shown. The alignment regions with the identity of >80% are in green color, while those with the identity of <80% are in orange color. [file DataSheet_1.pdf]

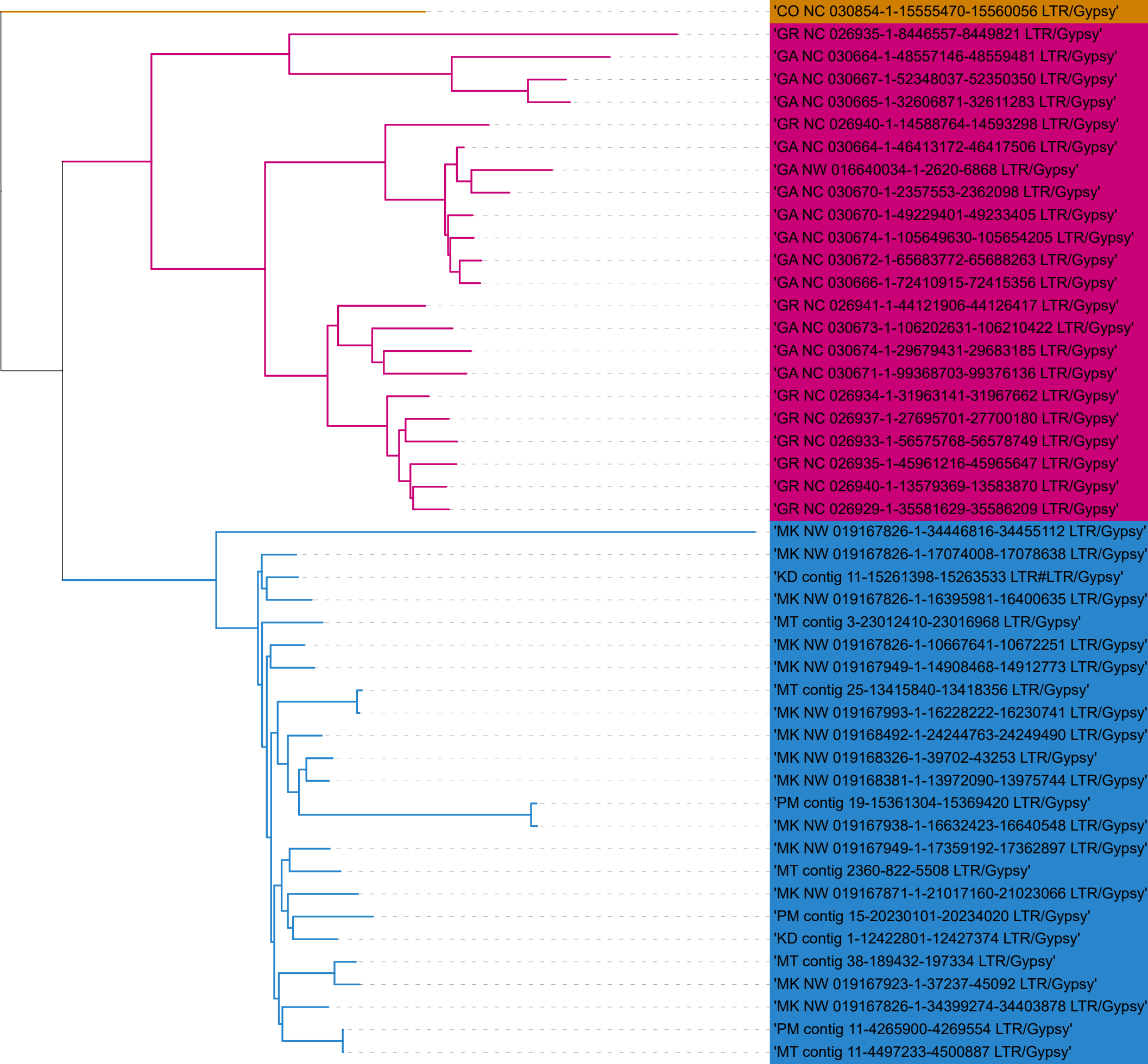

Supplement: Supplementary Figure 2 — The phylogenetic tree of LTR/Gypsy elements in the studied Malvales genomes. The LTR/Gypsy elements of cacao (CO), cottons (GA = G. arboreum and GR = G. raimondii) and durians are shown in orange, pink and blue colors. [file DataSheet_2.pdf]

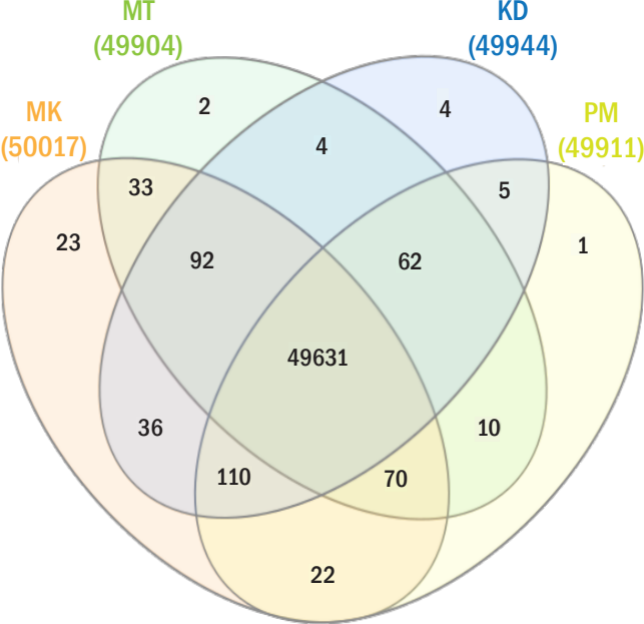

Supplement: Supplementary Figure 3 — The numbers of DNA mapped genes. The Venn diagram showed the numbers of genes on the draft pangenome that are mapped by DNA reads of each durian cultivar. [file DataSheet_3.pdf]
